# Predicting Cu and Zn sorption capacity of biochar from feedstock C/N ratio and pyrolysis temperature

**DOI:** 10.1007/s11356-017-1047-2

**Published:** 2017-12-29

**Authors:** Alfonso Rodríguez-Vila, Heather Selwyn-Smith, Laurretta Enunwa, Isla Smail, Emma F. Covelo, Tom Sizmur

**Affiliations:** 1 Department of Geography and Environmental Science, University of Reading, Reading, UK; 2 Department of Plant Biology and Soil Science, University of Vigo, Vigo, Spain; 3 The Coal Authority, Mansfield, UK

**Keywords:** Biochar, Metal, Pyrolysis temperature, Sorption capacity, Feedstock material, C/N ratio

## Abstract

Biochars have been proposed for remediation of metal-contaminated water due to their low cost, high surface area and high sorption capacity for metals. However, there is a lack of understanding over how feedstock material and pyrolysis conditions contribute to the metal sorption capacity of biochar. We produced biochars from 10 different organic materials by pyrolysing at 450 °C and a further 10 biochars from cedar wood by pyrolysing at 50 °C intervals (250–700 °C). Batch sorption experiments were conducted to derive the maximum Cu and Zn sorption capacity of each biochar. The results revealed an exponential relationship between Cu and Zn sorption capacity and the feedstock C/N ratio and a sigmoidal relationship between the pyrolysis temperature and the maximum Cu and Zn sorption capacity. FTIR analysis revealed that as temperature increased, the abundance of functional groups reduced. We conclude that the high sorption capacity of high temperature biochars is due to an electrostatic attraction between positively charged Cu and Zn ions and delocalised pi-electrons on the greater surface area of these biochars. These findings demonstrate a method for predicting the maximum sorption capacity of a biochar based on the feedstock C/N ratio and the pyrolysis temperature.

**Electronic supplementary material:**

The online version of this article (10.1007/s11356-017-1047-2) contains supplementary material, which is available to authorized users.

## Introduction

Metal contamination of surface water by mine water discharged from abandoned metal mines represents an important problem throughout the world due to its impact on freshwater and estuarine ecology and the safety of drinking water (Byrne et al. 2012). In England and Wales, 5% of rivers are at risk of failing to meet their EU Water Framework Directive targets of good chemical and ecological status because of the impact of abandoned metalliferous mines that discharge metals (EA 2008; EA 2012; Johnston and Rolley 2008), of which zinc (Zn) and copper (Cu) are among those elements of greatest concern due to their high prevalence and solubility. Active water treatment technologies such as ion exchange, electro-coagulation, membrane filtration, packed-bed filtration and precipitation have high operation costs and sludge disposal problems (Inyang et al. 2012). These disadvantages increase the need to develop passive and low-cost water treatments for Cu and Zn remediation of mine water.

The removal of metals has become one of the main focuses of research on the application of biochar for water treatment (Tan et al. 2015). Biochar is a carbon-rich material, derived from the pyrolysis of biomass in the absence of oxygen (Beesley et al. 2011). It has a high surface area, a considerable negative charge and a strong affinity for cations in water. These properties have led to it being suggested as a potential candidate to remove Cu and Zn from aqueous solutions (Wang et al. 2015).

Several mechanisms play a role in controlling the removal of heavy metals from aqueous solutions by biochar, but specific sorption (chemisorption) and non-specific sorption (physisorption) are the primary mechanisms for biochars with a low mineral ash content. Chemisorption occurs due to a chemical bond being formed between the adsorbate (metal cations, e.g. Cu and Zn) and the adsorbent (functional groups on the biochar surface, e.g. hydroxyl, carboxyl, phenolic). Prevalence of this mechanism in biochar-metal sorption is dependent on the solution pH and point of zero charge of biochar (Dong et al. 2011; Mukherjee et al. 2011). Physisorption is a mechanism of electrostatic (cation—π) interactions between metal cations in solution and the negative charge generated on the surface of the biochar due to delocalised π-electrons on aromatic structures (Harvey et al. 2011). It involves the removal of heavy metals by diffusional movement of metal ions into sorbent pores without the formation of chemical bonds.

Pyrolysis is a thermochemical process that produces biochar as a solid product (Inyang and Dickenson 2015), and the feedstock biomass can be combusted at several different temperatures (Hussain et al. 2016). Theoretically, biochar could be produced from any organic material (Lehmann and Joseph 2015). Agricultural and forest residues, industrial by-products and wastes, municipal solid waste materials and non-conventional materials, such as waste tires, papers and bones, are some of the wide range feedstock materials used for biochar production (Inyang et al. 2016).

The feedstock material and pyrolysis temperature have effects on the properties of biochar and thus influence the sorption efficiency (Sun et al. 2014). The pyrolysis temperature is a key factor that greatly influences the nature of biochar by significantly altering the structure and its sorption properties (Chen et al. 2012). Kim et al. (2013) reported that the pyrolysis temperature significantly influenced the structural, elemental and morphological properties of biochar. As a result, pH and surface area of biochar increased greatly at pyrolysis temperatures > 500 °C, resulting in the increase of sorption capacity with increasing pyrolysis temperature. Increasing pyrolysis temperature favours high surface areas and pore volumes in biochars (Inyang et al. 2016). Sorption of aromatic contaminants increases with increasing pyrolysis temperature, due to an increase in the accessible micro-pore volume and thus a greater surface area (Chen et al. 2008). The sorption capacity of biochar is also significantly influenced by the mineral composition of feedstock material (Tan et al. 2015). Xu et al. (2013a) compared the removal effect of Pb, Cu, Zn and Cd from aqueous solutions by rice husk biochar and dairy manure biochar. The results indicated that the removal ability varied with different biochar feedstock sources and the mineral components play an important role in the sorption capacity of biochar. Given the same pyrolysis conditions, the metal sorption capacity of biochars varies considerably when even very similar types of feedstock materials are compared (Sun et al. 2014). Sun et al. (2014) reported that biochars derived from different crop straws (corn straw, cotton straw, wheat straw and rice straw) exhibit distinct sorption capacities due to various surface characteristics.

There is a lack of mechanistic knowledge concerning the influence of the feedstock material and pyrolysis temperature on the sorption capacity of the resulting biochar. This lack of understanding prevents us from predicting the performance of a biochar, and thus prevents us from producing custom-designed biochars under defined conditions for specific applications, without arbitrary experimentation.

This study aims to demonstrate a method to predict Cu and Zn sorption capacity of biochar based on feedstock material and pyrolysis temperature. Biochars from 10 different organic materials were produced by pyrolysing at 450 °C, and then further 10 biochars were produced from one organic material (cedar wood) by pyrolysing at 10 different temperatures, increasing at 50 °C intervals from 250 to 700 °C. Batch sorption experiments were conducted to construct Cu and Zn sorption isotherms and then fitted to Langmuir models to derive the maximum sorption capacity of each biochar. We demonstrate in this paper a method for predicting the maximum sorption capacity of biochar based on a simple property of the feedstock material and the temperature of pyrolysis.

## Materials and methods

### Biochar production

Biochars were produced from ten different feedstock materials: cedar wood, greenwaste compost, pistachio nut shells, horse chestnut leaves, conifer bark, chicken manure, farmyard manure, whitewood spruce, pine stripwood and bamboo canes. Details of where these materials were obtained are given in the supporting information (Table SI-1). Prior to pyrolysis, the pistachio nut shells were soaked in warm water for 6 h to reduce the salt content, following Komnitsas et al. (2015). Woody materials were chipped to reduce their size. The feedstock materials were pyrolysed at 450 °C for 1 h in a Gallenkamp Muffle Furnace inside tins with a small hole in cut into the lid to prevent pressure buildup. Cedar wood was also pyrolysed at temperatures from 250 to 700 °C inclusive, at 50 °C intervals. The furnace was allowed to cool overnight before removing the biochar. All biochars were then ground to a fine powder using a TEMA mill (Laboratory Disc Mill T100ACH).

### Biochar characterisation

Total C and N content of biochars and feedstock materials was determined in triplicate 4 mg samples with a Thermo Scientific Flash 2000 Organic Elemental Analyser alongside one blank every 20 samples and a 5 mg in-house reference (97% ± 2.9% recovery for N) traceable to GBW07412 approved by State Bureau of Technical Supervision, The People’s Republic of China. The instrument was calibrated with 1 and 3 mg samples of an aspartic acid standard.

Attenuated total reflectance Fourier transform infrared (ATR FTIR) spectra were obtained for all the biochar samples using a PerkinElmer Spectrum 100 FTIR spectrometer equipped with a universal ATR sampling accessory. The spectra were obtained in the range from 550 to 4000 cm^−1^, a resolution of 4 cm^−1^ and 32 scans. Two subsamples were analysed for each biochar and the average spectra reported. Peaks were assigned to functional groups, primarily following Keiluweit et al. (2010) and Hina et al. (2010).

### Batch sorption experiment

Batch sorption was carried out using a variation of a published method (Kim et al. 2013). Nine Cu and Zn solutions (20, 50, 100, 150, 200, 300, 500, 1000 and 5000 mg Cu L^−1^ or mg Zn L^−1^) were prepared in > 18.2 MΩ.cm water by serial dilution of a 5000-mg L^−1^ stock solution made from copper sulphate (CuSO_4_) or zinc sulphate (ZnSO_4_). Dry-powdered biochar samples were weighed in triplicate into 50-mL centrifuge tubes (1 g ± 0.05 g), and 30 mL of Cu or Zn solution was added to each sample alongside 3 blank tubes run without biochar at each concentration. Samples were then placed on an end-over-end shaker at 30 rpm for 24 h followed by centrifugation at 2500 rpm for 15 min (MSS MISTRAL 3000i) at 20 °C, and filtering through a Whatman no. 5 filter paper. A 10-mL sample was then acidified with 5% HNO_3_ and analysed using ICP-OES (Perkin Elmer Optima 7300 DV Inductively Coupled Plasma-Optical Emission Spectrometer).

### Isotherm fitting

The concentration of Zn or Cu sorbed to the biochar was calculated using the following equation:

$$ Cs=\frac{\left( Ci- Caq\right)\times V}{Sm} $$

where *Cs* = concentration on biochar (mg g^−1^), *Sm* = mass of the biochar (g), *Ci* = Cu or Zn concentration measured in the blank tubes (mg L^−1^), *Caq* = Cu or Zn concentration measured in the solution after sorption (mg L^−1^) and *V* = solution volume (L).

Sorption data was then fit to a Langmuir sorption isotherm:

$$ \frac{Cs}{Caq}=\frac{b\  Csm}{1+ Caq\ b} $$

where b is the constant and *Csm* = maximum sorption capacity of Cu or Zn on the biochar (mg g^−1^).

### Zn sorption to biochar from mine water samples

Contaminated mine water solutions were collected from two different mine locations: a circumneutral mine water collected from a former lead mine site in North Pennine Orefield at Cumbria in the north-west (NW) of England and an acid mine drainage (AMD) mine water collected from a former lead and barytes mine at Devon in the south-west (SW) of England. At both sites, the mine waters are flowing under gravity from disused mine workings. The sampling points were immediately downstream of the adits, shortly after the water came into contact with the atmosphere. The mine waters were used to investigate the sorption of zinc from complex solutions.

Each water sample was analysed in triplicate for pH, major and trace elements, anions and dissolved organic carbon after passing samples through 0.45-μm cellulose nitrate membrane filters. pH was determined using a Hanna portable pH meter. Dissolved major and trace elements in solution were measured with ICP-OES after acidifying with 5% HNO_3_. Dissolved anions were analysed with a Dionex DX-500 ion chromatograph. Dissolved organic carbon and inorganic carbon were analysed using a Shimadzu Total Organic Carbon analyser. This data is given in Table 1.

**Table 1:** Concentrations of dissolved elements, dissolved anions, dissolved organic (DOC) and dissolved inorganic (DIC) carbon and pH of the mine water samples (mean ± S.E. *n* = 3)

	Mine water from NW England (circumneutral)	Mine water from SW England (AMD)
pH	7.3 ± 0.10	3.2 ± 0.03
Zn (mg L^−1^)	8.98 ± 0.07	14.16 ± 0.04
Cd (mg L^−1^)	0.01 ± 0.00	0.04 ± 0.00
Cu (mg L^−1^)	< 0.0083^a^	0.01 ± 0.00
Fe (mg L^−1^)	< 0.0008^a^	36.24 ± 0.51
Pb (mg L^−1^)	< 0.0020^a^	2.33 ± 0.02
Cl^−^ (mg L^−1^)	1.40 ± 0.24	5.11 ± 0.22
SO_4_^2−^ (mg L^−1^)	35.22 ± 6.54	49.32 ± 2.50
DOC (mg L^−1^)	1.34 ± 0.23	0.80 ± 0.50
DIC (mg L^−1^)	34.02 ± 0.11	2.46 ± 0.19

*DOC* dissolved organic carbon, *DIC* dissolved inorganic carbon, *AMD* acid mine drainage

^a^Detection limit

The sorption of Zn from the two mine waters was determined for three selected biochars pyrolysed at 450 °C from cedar wood, greenwaste compost and pistachio nut shells. The sorption of only Zn from these mine water samples was assessed because Cu was below the limits of detection in the circumneutral mine water and close to these limits in the AMD mine water. Batch sorption was carried out by shaking 2 g biochar with 60 mL of each mine water sample for 24 h on an end-over-end shaker at 30 rpm in triplicate. This was followed by centrifugation at 2500 rpm for 15 min (MSS MISTRAL 3000i) at 20 °C, and filtering through a Whatman no. 5 filter paper. A 10-mL sample was then acidified with 5% HNO_3_ and analysed using ICP-OES.

### Statistical analysis

All statistical analyses were carried out using Minitab 17.0, including analysis of variance (ANOVA) to calculate the difference in means between the Zn or Cu sorbed by each biochar sample from the different mine water solutions. Tukey’s test was used for multiple comparisons. All means are reported ± standard error (S.E.). Experimental data was fit to non-linear models (as described below) using Microsoft Excel Solver.

## Results and discussion

### The abundance of functional groups decreases with increasing pyrolysis temperature

**Fig. 1:**
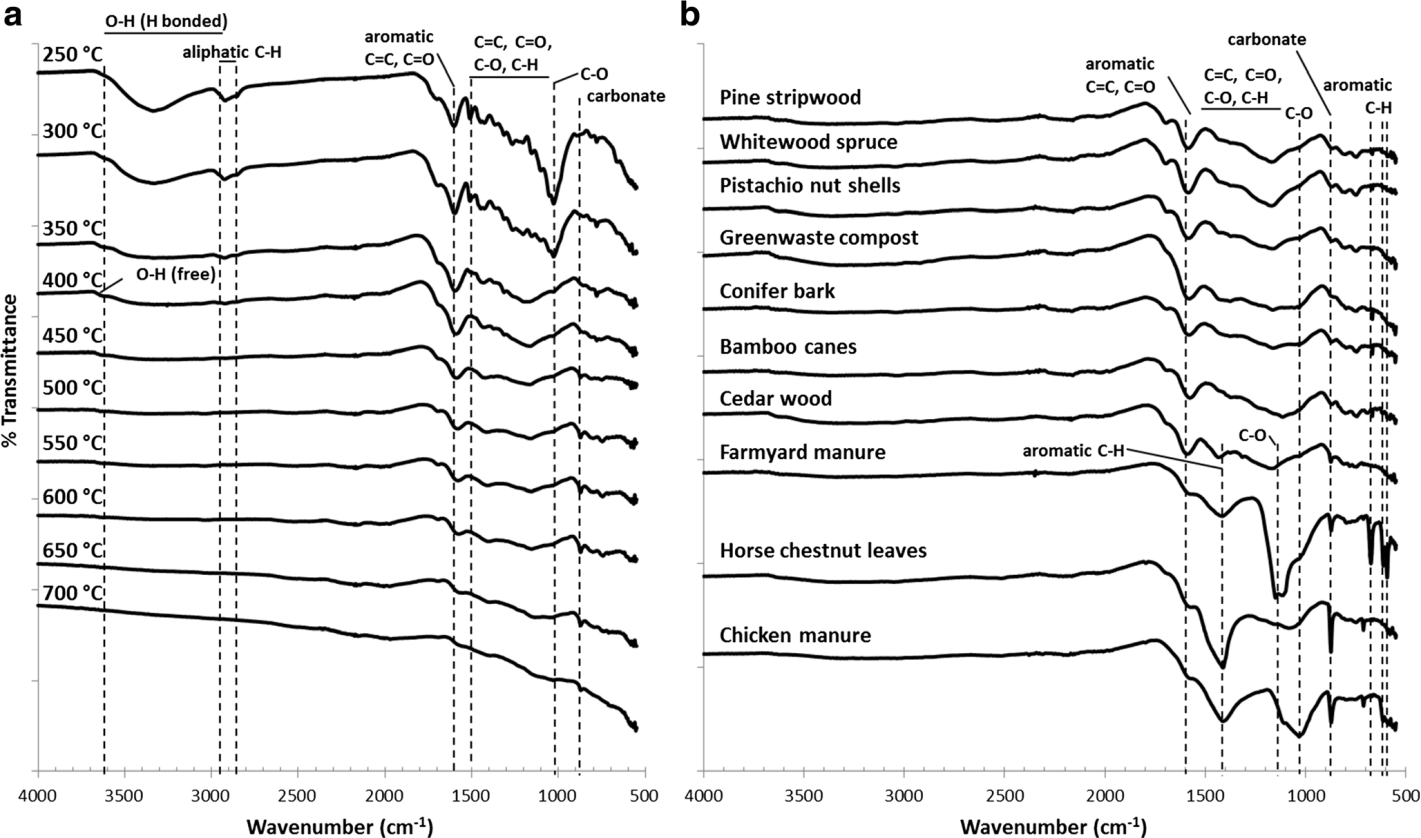
Stacked ATR FTIR spectra of biochars 250–700 °C (**a**) and biochars from the different feedstock materials (**b**)

The FTIR spectra of cedar wood biochar pyrolysed at temperatures 250 to 700 °C and biochars of 10 different feedstock materials pyrolysed at 450 °C is shown in Fig. 1a and b, respectively. With increasing pyrolysis temperature, the overall intensity of the infrared spectrum decreases along with the loss of functional groups, in agreement with Mochidzuki et al. (2003). There are multiple troughs in the 1500–1000 cm^−1^ region representing C=C, C=O, C–O and C–H groups that are less prominent as the pyrolysis temperature increases. The absorbance of the dominant C–O stretch near 1030 cm^−1^ can be assigned to cellulose, hemicellulose and lignin (Keiluweit et al. 2010) and disappears above 300 °C, suggesting the destruction or remarkable reduction of these structures (Zhang et al. 2011) in agreement with Labbé et al. (2006), Uchimiya et al. (2011) and Xiao et al. (2014). The trough at 3200–3500 cm^−1^ is associated with hydrogen bonded O–H stretching of water molecules and the band between 2950 and 2850 cm^−1^ can be attributed to the aliphatic C–H stretching (Keiluweit et al. 2010), both of which decrease as pyrolysis temperature increases and are not observable at 450 °C due to driving off of integral moisture and the volatilisation of aliphatic carbon.

Heating to temperatures ≥ 350 °C resulted in significant changes to the FTIR spectra (Fig. 1a) indicating substantial chemical transformations. There is a decrease in the intensity of the band at 1600 cm^−1^, which is indicative of aromatic C=C and C=O stretching (Chen et al. 2008; Hina et al. 2010; Keiluweit et al. 2010), with the increasing temperatures (Fig. 1a), but not to the same extent as the other bands. There is a decreased intensity and progressive coalescence of various carbohydrate and lignin derived signals (Baldock and Smernik 2002) in the 1500–1000 cm^−1^ region and there is an increase in intensity of the trough around 875 cm^−1^, associated with carbonate (Hina et al. 2010).

The spectra of pine stripwood, whitewood spruce, pistachio nut shells, greenwaste compost, conifer bark, bamboo canes and cedar wood biochars pyrolysed at 450 °C were similar (Fig. 1b). These biochars were characterised by a clear C=C and/or C=O trough at 1600 cm^−1^ (Hina et al. 2010; Keiluweit et al. 2010) that are due to the aromatic groups in lignin (Komnitsas et al. 2015). Clear differences were observed between these spectra and the spectra of farmyard manure, horse chestnut leaves and chicken manure biochars (Fig. 1b). The latter show an increase in intensity of the trough around 1400 cm^−1^ that can be assigned to aromatic ring vibrations combined with C–H in plane deformation (Hina et al. 2010) and a narrow band at 875 cm^−1^, associated with carbonate (Hina et al. 2010). Chicken manure and farmyard manure biochars showed large troughs at 1030 and 1150 cm^−1^, both assigned to C–O stretching. Farmyard manure revealed several troughs at 675, 615 and 595 cm^−1^, likely associated with aromatic C–H (Gusiatin et al. 2016; Inyang et al. 2010; Inyang et al. 2012).

### Cu and Zn sorption capacity increased with increasing biochar pyrolysis temperature

**Fig. 2:**
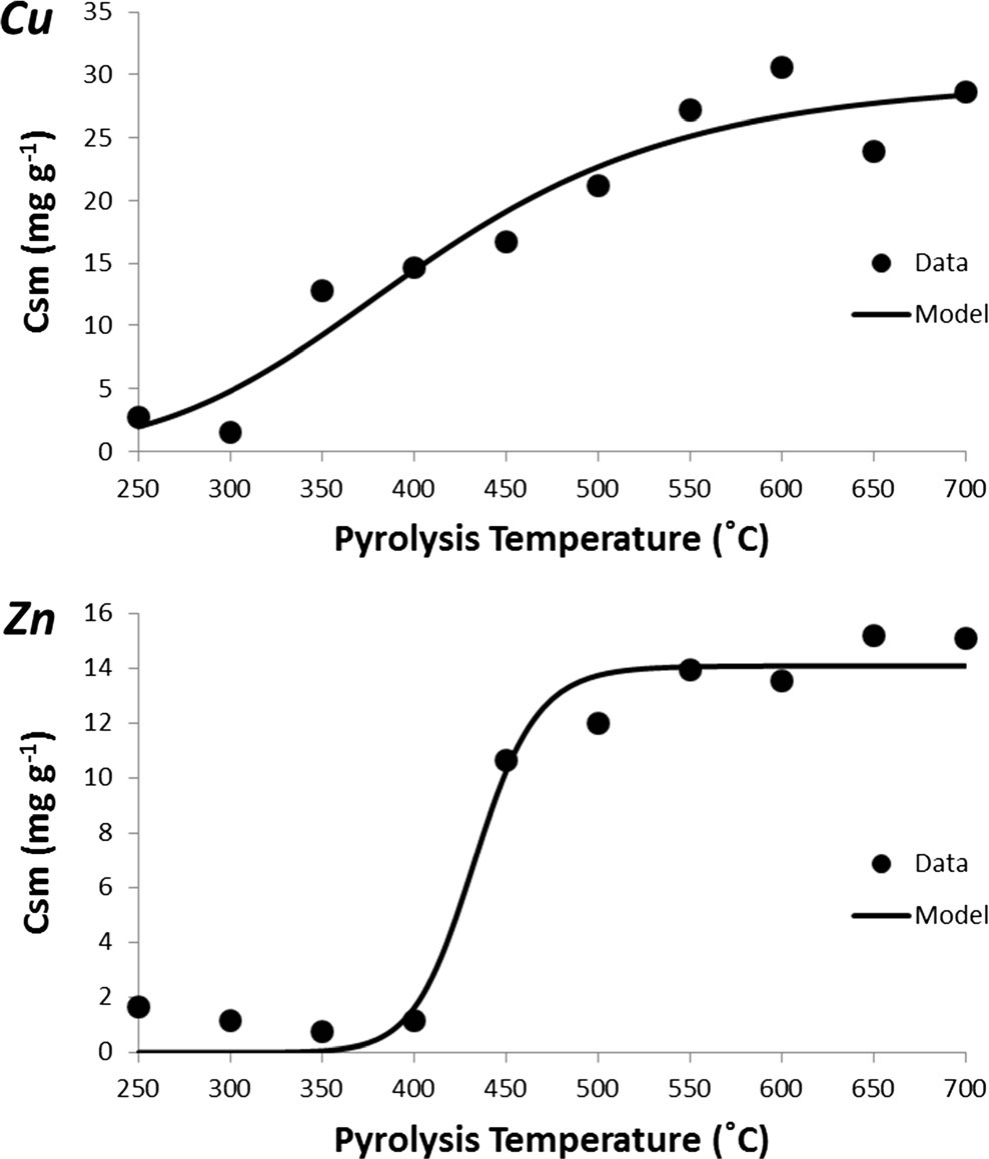
Maximum Cu and Zn sorption capacities (*Csm*) of cedar wood biochars plotted against their pyrolysis temperatures (250 to 700 °C) and fitted to the sigmoidal model described in Section Cu and Zn sorption capacity increased with increasing biochar pyrolysis temperature.

The Cu and Zn sorption isotherms for biochars produced at pyrolysis temperatures 250 to 700 °C are presented in the supporting information (Fig. SI-1 and SI-2, respectively). Maximum sorption (*Csm*) was derived for each biochar using the Langmuir fits (Table 2). The relationship between pyrolysis temperature of the biochar and the maximum Cu or Zn sorption capacity (*Csm*) was fit to the following sigmoidal curve (Fig. 2):

$$ Csm=m\ \frac{PT^n}{PT^n+{k}^n} $$

**Table 2:** Isotherm parameters (*Csm* = maximum sorption capacity (mg g^−1^), *b* = constant) and goodness of fit (*R*^2^) of a Langmuir model to describe Cu and Zn sorption onto biochars produced from Cedar wood at different pyrolysis temperatures and from different feedstock materials at 450 °C

Pyrolysis temperature (°C)	Feedstock material	Langmuir model (Cu)			Langmuir model (Zn)		
		*Csm*	*b*	R^2^	Csm	b	R^2^
250	Cedar wood	2.73	0.026	1.00	1.65	0.043	0.95
300	Cedar wood	1.50	0.026	0.94	1.14	0.106	0.96
350	Cedar wood	12.80	0.007	0.20	0.75	0.191	0.30
400	Cedar wood	14.66	0.016	0.54	1.17	0.188	0.99
450	Cedar wood	16.72	0.063	0.97	10.64	0.010	0.97
500	Cedar wood	21.23	0.151	0.99	12.01	0.097	0.85
550	Cedar wood	27.17	0.302	1.00	13.93	0.092	0.77
600	Cedar wood	30.58	0.321	0.99	13.51	0.181	0.88
650	Cedar wood	23.92	0.524	0.99	15.20	0.116	0.84
700	Cedar wood	28.65	0.496	0.99	15.06	0.140	0.86
450	Pine stripwood	0.82	3.527	0.77	0.59	0.102	0.00
450	Whitewood spruce	1.03	0.848	0.06	0.81	0.079	0.87
450	Pistachio nut shells	2.98	1.389	0.24	2.91	0.068	0.64
450	Greenwaste compost	6.55	2.475	0.99	5.18	0.295	0.94
450	Conifer bark	7.86	0.101	0.99	5.87	0.025	0.92
450	Bamboo canes	9.36	1.304	0.93	6.65	0.205	1.00
450	Cedar wood	16.72	0.063	0.97	10.64	0.010	0.97
450	Farmyard manure	35.84	2.250	0.94	26.04	0.202	0.79
450	Horse chestnut leaves	56.50	0.371	0.87	35.21	0.028	0.77
450	Chicken manure	81.30	1.398	0.95	31.95	6.521	0.95

where *PT* = pyrolysis temperature and m, k and n are constants. *m* is the maximum *Csm* and *k* is the pyrolysis temperature at which *Csm* = *m*/2. As *n* increases, the sigmoidal shape becomes more pronounced.

The sorption of Cu and Zn onto biochars was found to generally increase with increasing biochar pyrolysis temperature (Fig. 2) in agreement with Li et al. (2015) and Melo et al. (2013). The FTIR spectra revealed a general decrease in the abundance of functional groups on the surface of the biochar as the pyrolysis temperature increased (Fig. 1a), in agreement with Claoston et al. (2014), Pereira et al. (2015) and Uchimiya et al. (2011). Therefore, the increase in Cu and Zn sorption capacity with increasing pyrolysis temperature could not have been due to greater chemisorption on functional groups. The FTIR spectra also revealed that peaks associated with aromatic structures (e.g. 1600 cm^−1^) decreased in intensity with increasing pyrolysis temperature, although not as much as aliphatic peaks (e.g. 2950–2850 cm^−1^). Several authors have observed that the pore volumes and surface area of biochar increase with increasing pyrolysis temperature (Kloss et al. 2012; Suliman et al. 2016; Zhao et al. 2013). Suliman et al. (2016) show that as pyrolysis temperature increases, acidic functional groups decrease in abundance, and surface area (measured by N_2_ adsorption) increases between 500 and 600 °C with a sigmoidal relationship similar to the one we observed between pyrolysis temperature and Cu or Zn sorption. We therefore propose that the increase in sorption capacity observed is due to electrostatic attraction (physisorption) between Cu and Zn and the delocalised π-electrons of aromatic structures (Gomez-Eyles et al. 2013; Harvey et al. 2011; Sizmur et al. 2016) on a greater biochar surface area.

The relationship between the increase in metal sorption and increasing pyrolysis temperature was not similar for both Cu and Zn. Cu sorption capacity for most of the biochars was approximately double that of Zn and the shape of the increase with respect to pyrolysis temperature was different. While the increase occurs gradually between 350 °C and 550 °C for Cu, a large increase in Zn sorptive capacity can be seen from 400 to 500 °C, which may indicate a change in mechanism of metal sorption. This difference in the shape of the relationship between pyrolysis temperature and maximum sorption capacity for the two elements is reflected by the *n* constant of the sigmoidal model fits, which is a much higher value for the Zn fit (25.7) than the Cu fit (5.5). Since the difference cannot be explained by differences in the biochar, the difference must be due to the elements themselves. Although both Cu and Zn ions have the same charge, Cu is more electronegative than Zn and has a higher charge-to-radius ratio and thus Cu more readily forms bonds with functional groups on the surface of the biochar (McBride 1994). We therefore propose that the greater sorption of Cu ions on the surface of the biochar is due to specific sorption to surface functional groups which are gradually degraded with increasing pyrolysis temperature. Conversely, the sorption of Zn is primarily due to cation-π interactions with aromatic structures that only become available on the surface of the biochar above 400 °C due to the thermal cracking of cellulose and lignin (Xiao et al. 2014).

The sigmoidal fit indicated that the maximum possible Cu and Zn sorption capacity of the cedar wood biochar (at any temperature) was 29.8 and 14.1 mg g^−1^, respectively. The modelled pyrolysis temperatures at which sorption capacity is half the maximum possible (*k*) was 405 and 433 °C for Cu and Zn, respectively. This simple modelling approach allows biochar producers to identify the most efficient temperature to pyrolyse biochars to balance maximum sorption with minimum energy input.

### Cu and Zn sorption capacity increased with decreasing biochar feedstock C/N ratio

**Fig. 3:**
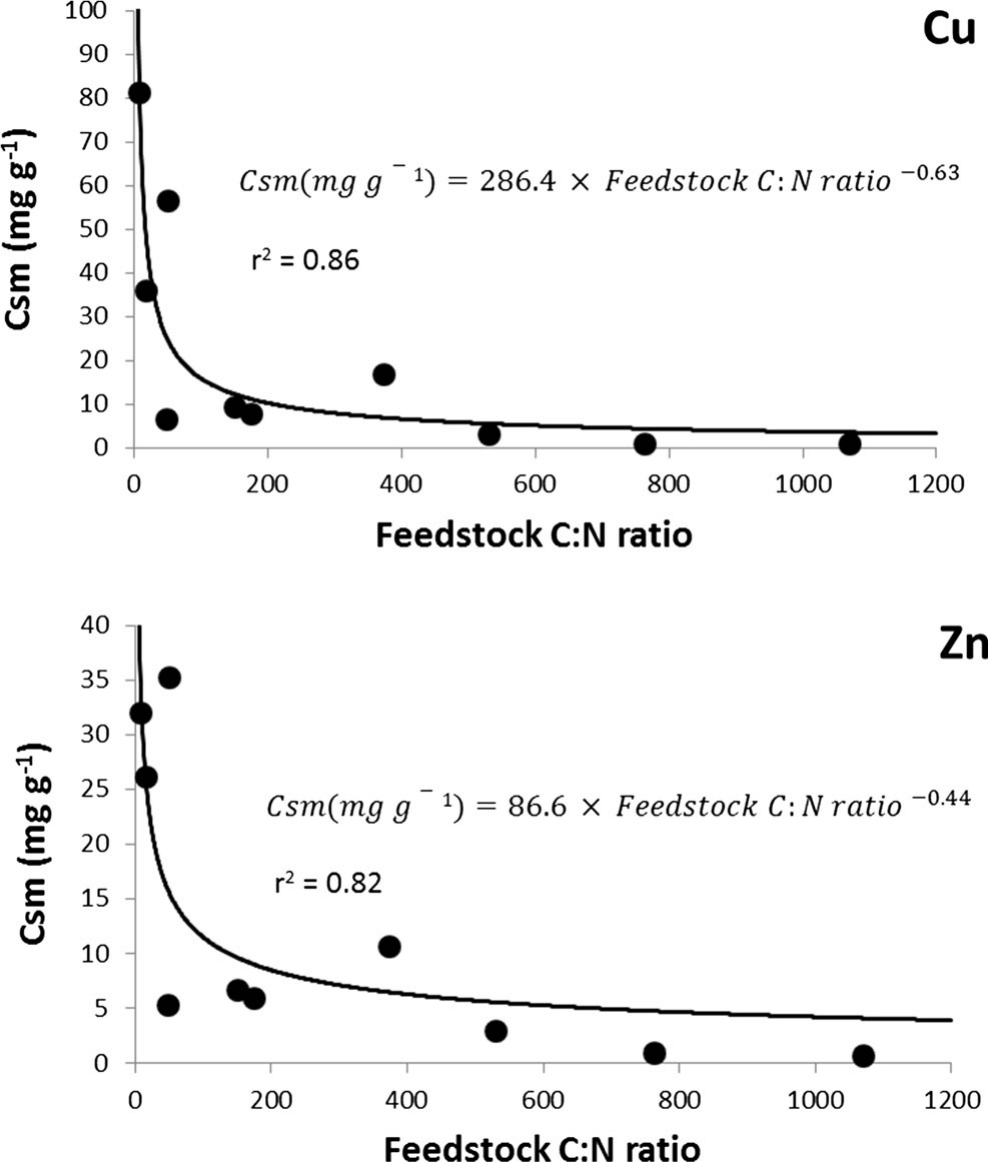
Maximum Cu and Zn sorption capacities (*Csm*) plotted against the C/N ratio of the feedstock materials of 10 biochars pyrolysed at 450 °C and fitted with a model describing exponential reduction in *Csm* as C/N ratio of the feedstock material increases

The Cu and Zn sorption isotherms for biochars produced from the different feedstock materials are presented in the supporting information (Fig. SI-3 and SI-4, respectively). Maximum sorption capacity (*Csm*) was derived for each biochar using the Langmuir fits (Table 2), plotted against biochar feedstock C/N ratio and presented in Fig. 3. The sorption capability of biochar was significantly influenced by the compositions of feedstock material, as observed by Sun et al. (2014). There is a general trend that Cu and Zn sorption increases exponentially with decreasing biochar feedstock C/N ratio. This relationship was similar for both Cu and Zn sorption, despite Cu sorption being approximately double that of Zn sorption. This observation implies that a single mechanism of sorption is affected by the C/N ratio of the biochar feedstock when biochars are pyrolysed at 450 °C. Organic materials with a high C/N ratio often contain high lignin and cellulose contents. The materials with the highest C/N ratios (pine stripwood, whitewood spruce and pistachio nut shells; Table 3) all have high lignin content. Feedstock materials with higher carbon content or lignin composition have previously been found to have lower sorption capacity if they contain very low nitrogen content (Komnitsas et al. 2015; Tan et al. 2015). Conversely, biochars produced from materials with low lignin content have greater microporosity which enhances metal sorption (Bogusz et al. 2015), and there is a negative relationship between surface area and the lignin content of feedstock materials (Sun et al. 2014). It therefore seems that materials with less woody biomass produce biochars with a greater surface area and a higher Cu and Zn sorption capacity. The higher sorption capacity of the manure biochars (farmyard manure and chicken manure) may be also attributed to the precipitation of phosphate and carbonated minerals (Cao et al. 2009; Xu et al. 2013b). This finding agrees with the idea that mineral elements in the biochar can serve as additional sorption sites (Uchimiya et al. 2010).

**Table 3:** Percentage of carbon and nitrogen content and C/N ratio in the different feedstock materials (mean ± S.E. *n* = 3)

Feedstock material	%C	%N	C/N ratio
Pine stripwood	46.3 ± 0.28	0.04 ± 0.00	1070 ± 74.3
Whitewood spruce	45.4 ± 0.08	0.06 ± 0.00	763 ± 34.0
Pistachio nut shells	45.8 ± 0.12	0.09 ± 0.01	530 ± 32.4
Greenwaste compost	48.4 ± 0.03	1.01 ± 0.01	48 ± 0.4
Conifer bark	46.1 ± 0.22	0.27 ± 0.03	175 ± 20.0
Bamboo canes	44.0 ± 0.47	0.29 ± 0.01	150 ± 8.6
Cedar wood	49.7 ± 0.38	0.13 ± 0.01	374 ± 19.6
Farmyard manure	32.5 ± 0.86	1.95 ± 0.03	17 ± 0.7
Horse chestnut leaves	46.1 ± 0.05	0.90 ± 0.00	51 ± 0.2
Chicken manure	36.4 ± 0.47	4.43 ± 0.03	8 ± 0.1

We found a significant positive correlation between the ratio of the % transmittance at 1600 and 1400 cm^−1^ of the FTIR spectra and the maximum Cu (*r*^2^ = 0.87, *p* < 0.001) and Zn (*r*^2^ = 0.93, *p* < 0.001) sorption capacity. While both bands (1600 and 1400 cm^−1^) are associated with aromatic structures, 1600 cm^−1^ is associated with both C=C stretching and C=O conjugated to the aromatic ring and 1400 cm^−1^ is associated with C–H plane deformation (Hina et al. 2010). It therefore seems that biochars with a high abundance of carbonyl (C=O) groups associated with aromatic structures (represented by 1600 cm^−1^) have a lower Cu and Zn sorption capacity than biochars with an overall high degree of aromaticity.

### The use of biochar to remove Zn from contaminated mine water

Of the three biochars selected for an assessment of Zn sorption from mine waters, the greenwaste compost-derived biochar sorbed the greatest concentration of Zn and the greatest proportion of Zn from both the AMD and the circumneutral mine waters (Table 4). Greenwaste compost biochar also had the lowest C/N ratio. These measurements made in real (complex) mine waters are in agreement with our observations that Zn sorption capacity increases exponentially with decreasing C/N ratio of biochar feedstocks (Fig. 3).

**Table 4:** Zinc sorption from an acid mine drainage (AMD) mine water and a circumneutral mine water by three biochars produced by pyrolysis at 450 °C. Data with different letters are statistically significantly different (*p* < 0.01) (mean ± S.E. *n* = 3)

Mine water	Biochar	C/N ratio of biochar feedstock	Zinc sorbed (mg g^−1^ of biochar)	Zinc sorbed (%)
Mine water from NW England (circumneutral)	Cedar wood	374 ± 19.6	0.029 ± 0.0007 ^b^	77.6 ± 0.6
	Greenwaste compost	48 ± 0.4	0.037 ± 0.0008 ^a^	98.5 ± 0.2
	Pistachio nut shells	530 ± 32.4	0.032 ± 0.0010 ^b^	84.2 ± 0.7
Mine water from SW England (AMD)	Cedar wood	374 ± 19.6	0.396 ± 0.0022 ^c,d^	92.7 ± 0.4
	Greenwaste compost	48 ± 0.4	0.426 ± 0.0038 ^c^	99.8 ± 0.0
	Pistachio nut shells	530 ± 32.4	0.353 ± 0.0046 ^d^	82.7 ± 0.7

*AMD* acid mine drainage

More Zn was sorbed by the biochar from the AMD mine water than the circumneutral mine water (Table 4), and this could have been because there was a greater concentration of Zn in the AMD water (14.16 mg L^−1^ compared to 8.98 mg L^−1^; Table 1), or it could have been because the AMD water had a lower pH. Biochars are often found to have a high pH (Yuan et al. 2011) and the cedar wood, greenwaste compost and pistachio nut shell biochars in this experiment had a pH of 7.9, 9.3 and 9.6, respectively (measured in water at 1:20 *w*/*v*). The lower pH in the cedar wood biochar may also explain why it does not perform as well in the circumneutral mine water as the pistachio nut shell biochar, despite having a lower C/N ratio. Greater sorption of metal cations to negative surface occurs at higher pH because there is less competition between H^+^ ions and the metal cations for either specific sorption sites on oxygenated functional groups (e.g. hydroxyl, carboxyl) or within the stern layer of electrostatically charged ions attracted to a surface. Chen et al. (2011) show that a biochar produced from a mix of hardwood species had a pH of 5.57 and did not sorb as much Cu and Zn as a biochar with a higher pH and Kolodyńska et al. (2012) reveal that the initial pH of the solution had a large influence on the efficiency of biochar for metal removal and identified an initial pH of 5.0 for optimum removal Cu and Zn by biochar. The pH of the biochar may therefore become an important factor determining the metal sorption capacity from mine water, especially when the mine water has a higher or similar pH to the biochar when competition for sorption sites becomes as important as the number of sites available.

## Conclusions

The results of this study revealed that the abundance of functional groups on the surface of the biochar decreased with increasing biochar pyrolysis temperature. Cu and Zn sorption capacity increased with increasing biochar pyrolysis temperature and with decreasing biochar feedstock C/N ratio. We conclude that the high sorption capacity of high temperature biochars produced from feedstocks with a low C/N ratio is due to an electrostatic attraction between the positively charged Cu and Zn ions and the delocalised π-electrons on aromatic structures on the greater surface area of these biochars. The sorption capacity of biochar may be also significantly influenced by the mineral composition of biochars derived from manures. These relationships between biochar production parameters (feedstock material, pyrolysis temperature) and the metal sorption capacity of the resulting biochar are the first step in creating a biochar sorption model that will enable us to predict and produce custom biochars for specific applications, without costly experimentation. However, when applying these biochars to real (complex) solutions, the chemistry of the solution may limit the potential of the biochar sorption capacity to be realised. We show that the pH of the biochar and the solution may be important factors determining the metal sorption capacity of biochar from mine water samples.

## Electronic supplementary material

ESM 1 (DOCX 221 kb)
