# Supplementary material for: Predicting Cu and Zn sorption capacity of biochar from feedstock C/N ratio and pyrolysis temperature
Source: Environ Sci Pollut Res Int. 2017 Dec 29;25(8):7730–9. doi: 10.1007/s11356-017-1047-2 (PMC5847629; doi:10.1007/s11356-017-1047-2)
Supplement: Supplementary file 1 — (DOCX 221 kb) [file 11356_2017_1047_MOESM1_ESM.docx]

**Supplementary material for:**

**Predicting Cu and Zn sorption capacity of biochar from feedstock C:N ratio and pyrolysis temperature**

Alfonso Rodríguez-Vila, Heather Selwyn-Smith, Laurretta Enunwa, Isla Smail, Emma F. Covelo and Tom Sizmur

Contents: One table and four figures

**Table SI-1** Sources of the different feedstock materials

| Feedstock material | Source |
| --- | --- |
| Cedar wood | Graveyard at St Paul’s Church, Wokingham |
| Greenwaste compost | B&Q hardware store |
| Pistachio nut shells | Tesco supermarket |
| Pine stripwood | Homebase garden centre |
| Whitewood spruce | Homebase garden centre |
| Conifer bark | Homebase garden centre |
| Horse chestnut leaves | University of Reading campus |
| Bamboo canes | Homebase garden centre |
| Farmyard manure | Homebase garden centre |
| Chicken manure | Homebase garden centre |


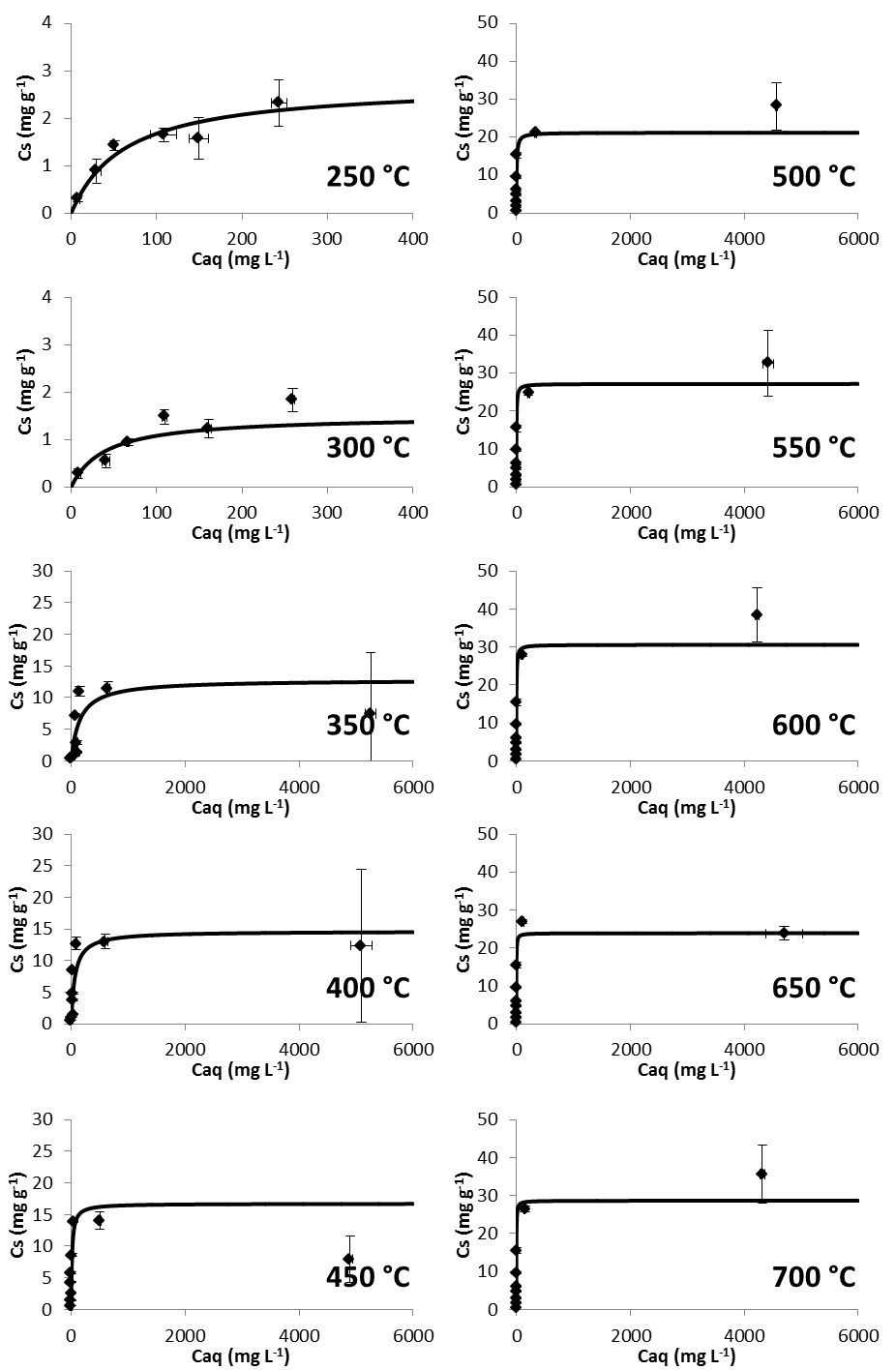


**Fig. SI-1** Cu sorption isotherms for biochars 250-700 °C fitted to Langmuir isotherms. Each data point is the average of three replicate measurements, error bars are standard errors of the mean

**
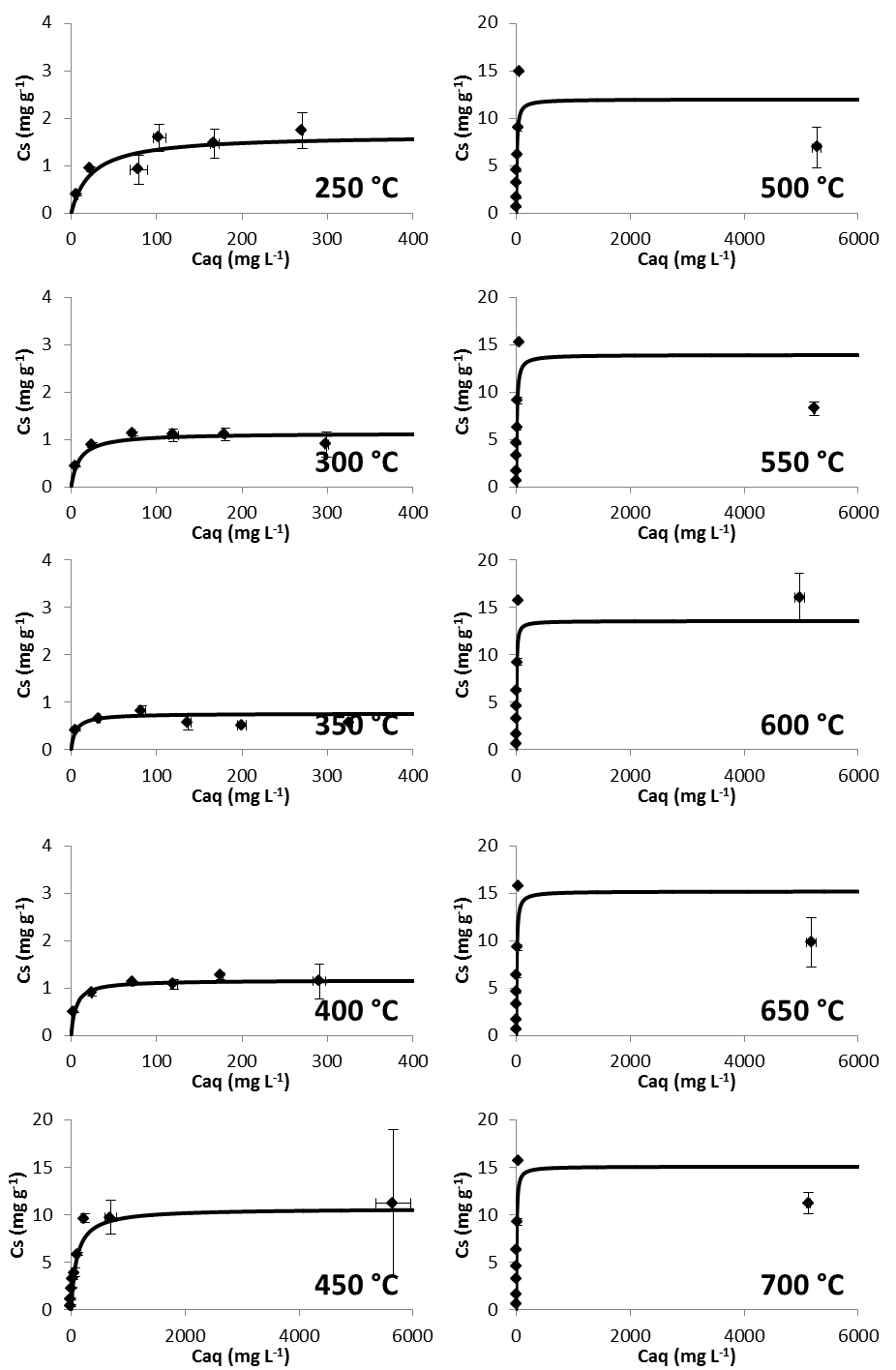
**

**Fig. SI-2** Zn sorption isotherms for biochars 250-700 °C fitted to Langmuir isotherms. Each data point is the average of three replicate measurements, error bars are standard errors of the mean

**
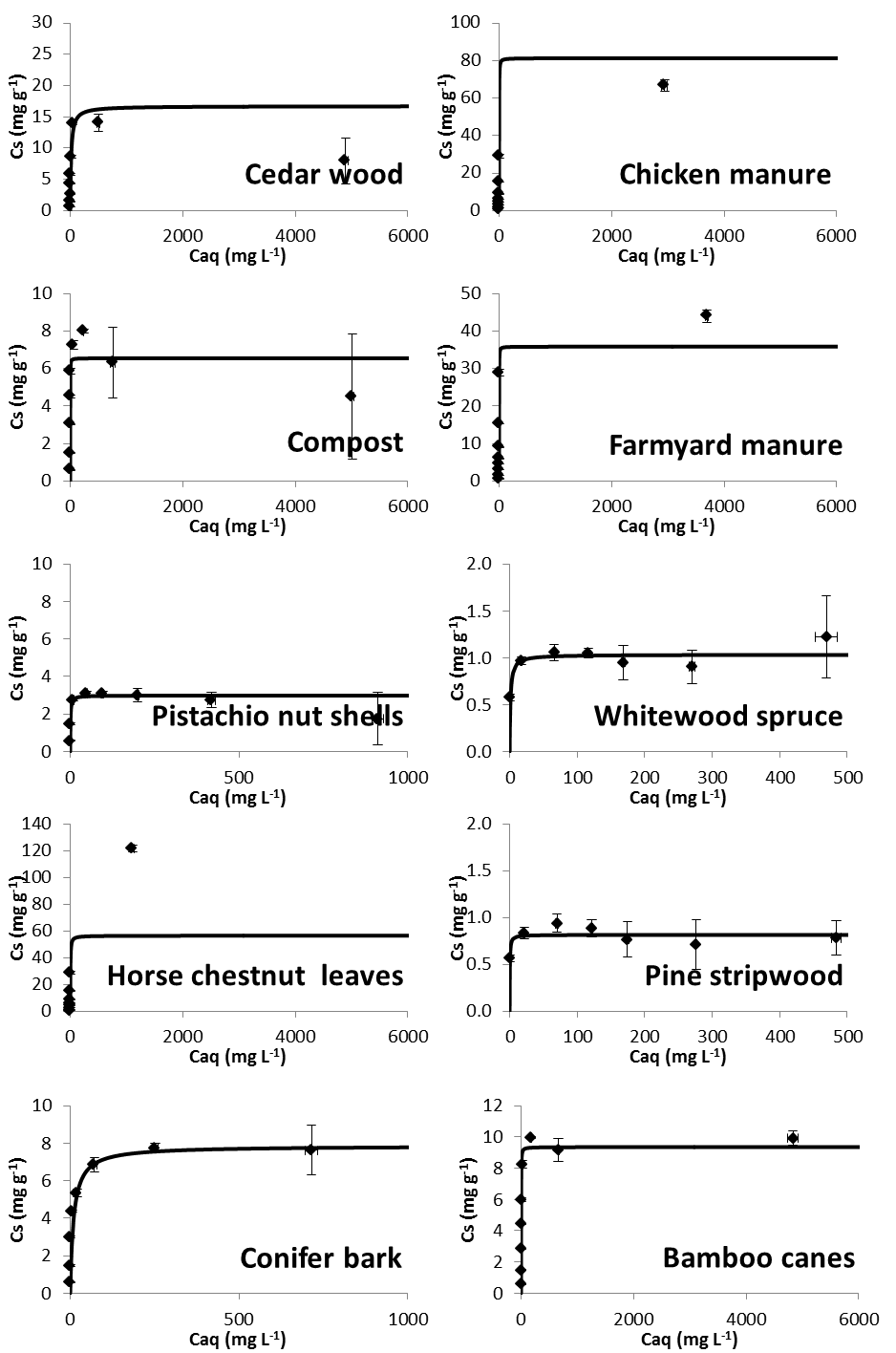
**

**Fig. SI-3** Cu sorption isotherms for biochars from the different feedstock materials fitted to Langmuir isotherms. Each data point is the average of three replicate measurements, error bars are standard errors of the mean

**
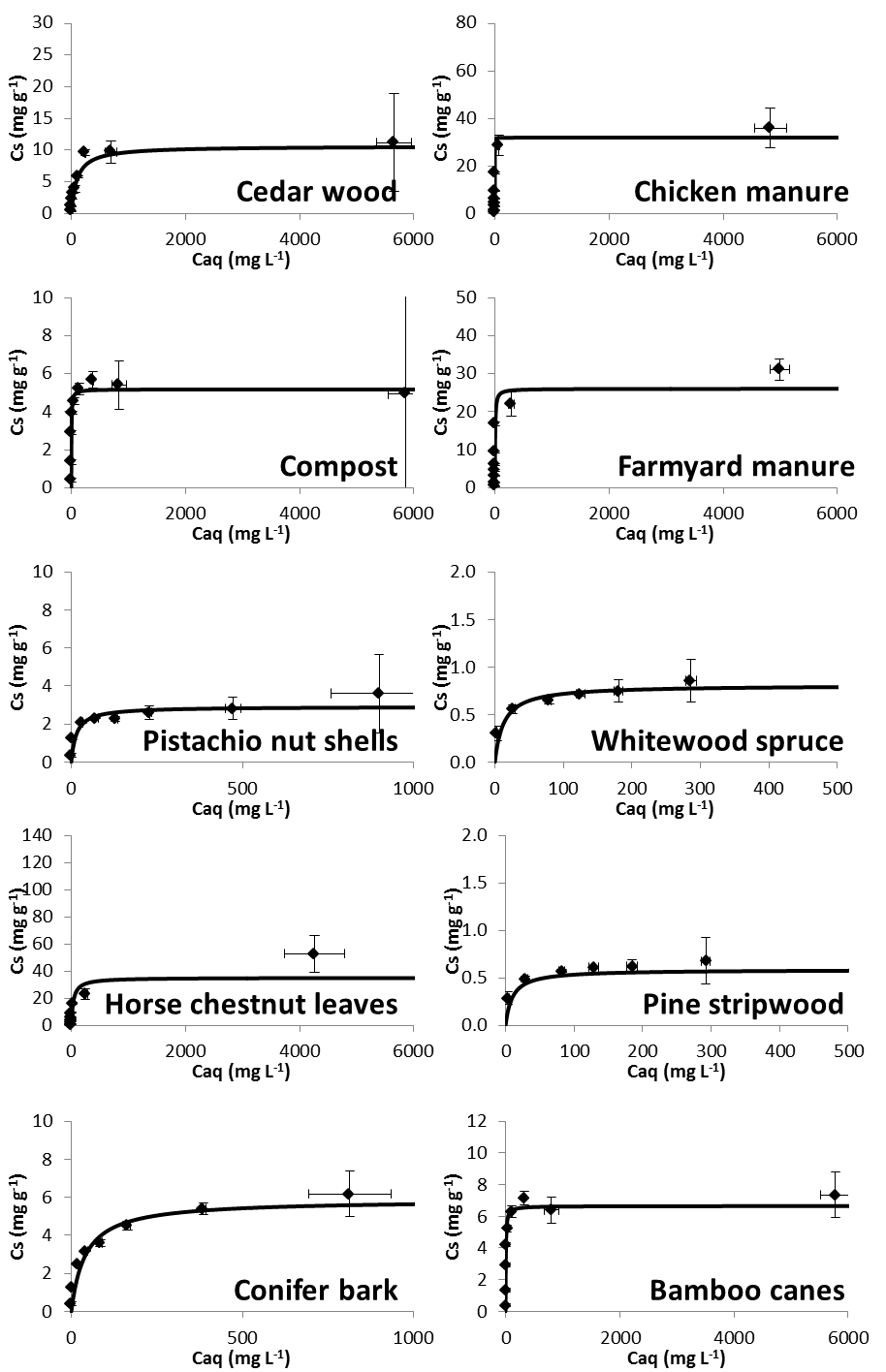
**

**Fig. SI-4** Zn sorption isotherms for biochars from the different feedstock materials fitted to Langmuir isotherms. Each data point is the average of three replicate measurements, error bars are standard errors of the mean
